# Visual perspective-taking in complex natural scenes

**DOI:** 10.1177/17470218211054474

**Published:** 2021-10-25

**Authors:** Paola Del Sette, Markus Bindemann, Heather J Ferguson

**Affiliations:** 1 Department of Brain and Behavioural Sciences, University of Pavia, Pavia, Italy; 2 School of Psychology, University of Kent, Canterbury, UK

**Keywords:** Perspective-taking, altercentric interference, cuing paradigm, scene perception

## Abstract

Studies of visual perspective-taking have shown that adults can rapidly and accurately compute their own and other peoples’ viewpoints, but they experience difficulties when the two perspectives are inconsistent. We tested whether these egocentric (i.e., interference from one’s own perspective) and altercentric biases (i.e., interference from another person’s perspective) persist in ecologically valid complex environments. Participants (*N* = 150) completed a dot-probe visual perspective-taking task, in which they verified the number of discs in natural scenes containing real people, first only according to their own perspective and then judging both their own and another person’s perspective. Results showed that the other person’s perspective did not disrupt self perspective-taking judgements when the other perspective was not explicitly prompted. In contrast, egocentric and altercentric biases were found when participants were prompted to switch between self and other perspectives. These findings suggest that altercentric visual perspective-taking can be activated spontaneously in complex real-world contexts, but is subject to both top-down and bottom-up influences, including explicit prompts or salient visual stimuli.

## Introduction

Visual perspective-taking (VPT) is a crucial component of our ability to understand and predict other’s mental states, and is linked with Theory of Mind (ToM). Research on this topic has examined two types of VPT that can be differentiated by whether or not they require a mental rotation into the spatial position of the other person (Michelon & Zacks, 2006; Surtees et al., 2013). Level-1 VPT assesses the ability to understand *what* someone else can see, while Level-2 VPT assesses the ability to adopt someone else’s spatial point of view to judge *how* he or she sees the visual stimulus. The current study focuses on Level-1 VPT and tests the degree to which observers automatically compute other people’s and their own visual perspectives within complex real-world environments.

Previous research has developed a task to investigate Level-1 VPT in which participants verified the number of discs in a three-dimensional (3D) room according to either their own or an avatar’s perspective (Samson et al., 2010). Importantly, the stimuli were manipulated so that the participant and the avatar either saw the same number of discs (i.e., their perspectives were consistent) or the participant and the avatar saw a different number of discs (i.e., their perspectives were inconsistent). Numerous studies investigating the processes underling VPT using this task have converged in finding two cognitive biases that influence performance: egocentric and altercentric biases (e.g., Capozzi et al., 2014; Catmur et al., 2016; Conway et al., 2017; Ferguson et al., 2017; Furlanetto et al., 2016; Gardner et al., 2018; Nielsen et al., 2015; Qureshi et al., 2010; Santiesteban et al., 2014). Egocentric interference reflects participants’ tendency to be slower and less accurate when they have to judge the avatar’s perspective and inhibit their own different visual perspective (i.e., other-inconsistent trials). Altercentric interference reflects participants’ tendency to be slower and less accurate when they have to judge their own perspective and inhibit the avatar’s different visual perspective (i.e., self-inconsistent trials). Together these results suggest that the brain cannot ignore the irrelevant perspective and that performance on this VPT task involves automatic or spontaneous calculation of self and other perspectives (Samson et al., 2010). While this pattern of effects is fairly robust across studies, there remains much debate in the literature on the social/cognitive mechanisms that underlie the altercentric effect. Some studies suggest that it reflects involuntary mentalising about the avatar’s perspective, but others suggest that domain-general attentional processes drive the effect as directional features of the avatar bias attention to one side of the screen or the other, and it is this conflict that has to be resolved on inconsistent trials (i.e., sub-mentalising; Cole et al., 2016, 2017; Heyes, 2014; Langton, 2018; Santiesteban et al., 2014).

The implicit mentalizing account for VPT is supported by research showing that faces capture attention among other distractors (e.g., Bindemann & Burton, 2008; Bindemann et al., 2007, 2010; Birmingham et al., 2009; Brown et al., 1997; Dupierrix et al., 2014; Farroni et al., 2005; Hershler & Hochstein, 2005; Olk & Garay-Vado, 2011), and that this attentional advantage enhances the cognitive efficiency of processing human forms (Mayer et al., 2015). This prominent role for faces in social perception could explain the altercentric bias found in VPT studies: since attention is automatically drawn to faces, it would be difficult to disengage from other people’s perspectives. In line with this implicit mentalising account, some researchers have shown that attentional effects are attenuated when the avatar was replaced by a non-social directional cue (Nielsen et al., 2015; Samson et al., 2010), Experiment 3; Schurz et al., 2015), or when the avatar’s awareness of their surroundings was compromised by an occlusion (e.g., a barrier or opaque goggles; Baker et al., 2016; Furlanetto et al., 2016). This claim has been further supported by eye-tracking data showing that participants attended to the scene differently when they were cued to judge self versus other perspectives although the directional features of the avatar were matched (Ferguson et al., 2017), and by studies showing that the extent to which observers experience interference from the self/other perspective is modulated by in/out-group associations with the avatar (Ferguson et al., 2018; Simpson & Todd, 2017; Todd et al., 2011). Overall, these studies support the involvement of implicit mentalizing of what others can see and suggest that the domain-general approach may be too reductive to fully explain VPT.

The domain-general view proposes that the consistency effect is driven by domain-general processes based on directional features of the avatar (Heyes, 2014; Santiesteban et al., 2014, 2017). In support of attentional processes rather than implicit mentalizing in VPT is evidence showing a comparable consistency effect when the avatar was replaced by a left- or right-facing arrow (e.g., Santiesteban et al., 2014), and studies that have shown attentional biases in line with the avatar’s gaze even when the avatar’s awareness was compromised (e.g., by a barrier or an “invisibility” telescope; Cole et al., 2016; Conway et al., 2017). Many of these studies have tested self-perspective trials in isolation, which enables an exploration of implicit altercentric interference without carry-over effects from explicit, non-automatic mentalising when self and other perspectives were probed. It is possible that *both* directional processes and implicit mentalizing underlie the altercentric effect in VPT, and that the degree to which observers experience interference from the other perspective is modulated by top-down processes that increase the salience of the avatar’s perspective and focus attention onto differences in mental states or altered gaze following, such as explicit instructions to track the other perspective (e.g., Ferguson et al., 2018; Gardner et al., 2018; Nielsen et al., 2015; O’Grady et al., 2020; Qureshi et al., 2010; Slessor et al., 2010).

Clearly, many of the experimental manipulations that have been used to date to disentangle mentalizing and attentional influences on VPT have been artificial in nature (e.g., goggles, telescopes, arrows, barriers). Moreover, all these previous studies have failed to account for the visual complexity of real-life natural environments by using unrealistic stimuli that increase the salience of the avatar’s perspective. First, previous studies have created stimuli that incorporate a single figure in a blank 3D computer-generated room, which makes the avatar and its directional features stand out. Comparing effects across previous studies suggests that increasing visual complexity of the scene (i.e., by adding barriers, a greater number of discs, a second avatar or an additional element) might reduce intrusions from the altercentric perspective (O’Grady et al., 2020). Moreover, increasing visual complexity has been shown to disrupt attention effects in face detection tasks; profile view faces are detected slower than frontal view faces when they are embedded in natural scenes, but are equally detectable when presented on plain backgrounds or in small centrally-located scenes (Bindemann & Lewis, 2013). Second, previous studies have used computer simulations of avatars instead of real people, which is likely to evoke differential processing and enhanced perceptual discrimination (de Borst & de Gelder, 2015). Third, most previous studies have placed the avatar in the centre of the scene/screen, preceded by a central fixation cross, which increases the salience of the avatar due to the central viewing tendency in scene perception (Bindemann, 2010; Bindemann et al., 2010) and by providing an additional cue to the avatar’s location (Bukowski et al., 2015; O’Grady et al., 2020). While these simpler stimuli have allowed researchers to maximise experimental control, it is unclear whether the interference effects reported in previous studies generalise to the more complex, naturalistic situations that we experience in real-life perspective-taking situations.

### The current study

We present a pre-registered study that addresses the limitations presented above by investigating the extent to which egocentric and altercentric biases persist when VPT is tested in complex real-world scenes. We adapted and extended the research by Samson et al. (2010) on Level-1 VPT by using photographs of real people in complex natural scenes, rather than computer simulations of avatars in blank rooms, and varying the position of the person in the scenes so that they could appear on the left, right, or centre of the scene. This design presents a more ecologically-valid paradigm than has been used previously.^1^ To further test the automaticity of altercentric interference effects, we first tested self-perspective trials in isolation, before mixing self- and other-perspective trials in the second half of the experiment.

Testing VPT in this paradigm using more naturalistic stimuli has the potential to shed new light on the mechanisms that underlie implicit mentalizing and domain-general processing. The implicit mentalizing account assumes that faces automatically capture attention, and therefore the process of verifying the other person’s perspective should be relatively easy. However, spontaneous mentalising processes are subject to limits when the cognitive demands of the task are increased (e.g., by increasing the number of discs to verify; see Apperly & Butterfill, 2009), and it is possible that the complex visual environments used here will be sufficient to disrupt spontaneous perspective-taking. Therefore, if complex natural scenes are efficiently managed by observers, this account would predict a replication of the patterns reported in Samson et al.’s (2010) study, with consistency effects on accuracy and response time measures for both self and other perspectives (i.e., reflecting altercentric and egocentric interference, respectively), and altercentric effects persisting even when the self-perspective was tested in isolation (i.e., participants were not explicitly prompted to infer the other person’s perspective). However, if the complex visual scenes exceed observers’ processing limits this would interfere with other perspective-taking, meaning that the altercentric effect would be diminished or eliminated. A purely attentional account is much clearer in predicting no altercentric effects when the self-perspective is tested in isolation, and that consistency effects would be reduced or eliminated due to distractors in the natural environment, which would compete for attention, reduce the salience of the person’s directional features (i.e., nose, eyes), and deplete general processing resources needed to verify the other person’s perspective.

## Method

All methodological procedures were pre-registered on the Open Science Framework (OSF) web pages (https://osf.io/jb3tc).

### Participants

A total of 157 participants from the University of Kent took part in the study. Seven participants were excluded from analyses due to poor overall accuracy (< 60%; *N* = 6) or slow reaction times (> 2.5 standard deviations above the mean; *N* = 1). Thus, the final sample consisted of 150 participants, of which 127 were female, *M*_age_ = 19.07, *SD* = 0.94, range = 18–24. All participants provided written consent for the current study. All participants were fluent English-speakers, had normal or corrected-to-normal vision, and had no history of neurological disorders, diagnoses of mental health or autism spectrum disorder. Participant consent was obtained according to the Declaration of Helsinki, and the Ethical Committee of the School of Psychology, University of Kent, approved the study.

### Materials

**Figure 1.**
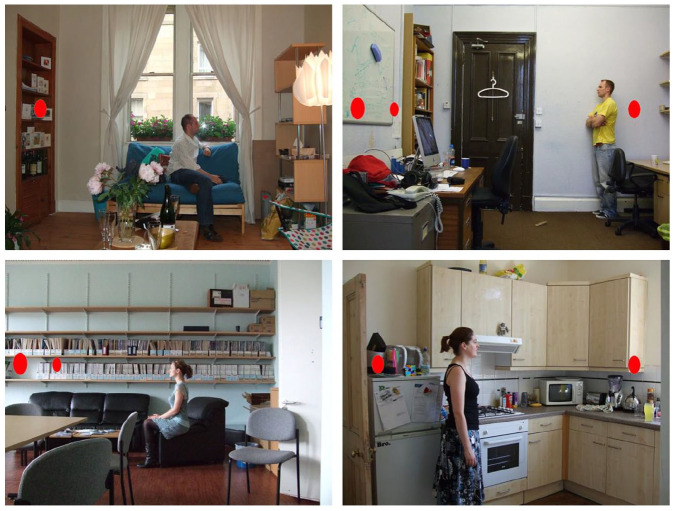
Examples of the visual stimuli, showing the variety of natural scenes and different configurations of discs to the left and right side of the scenes.

Participants completed a VPT task adapted from Samson et al. (2010). The visual stimuli included photographs of natural scenes taken from Bindemann et al. (2010), and depicted 34 different indoor and outdoor environments (e.g., living room, kitchen, or garden). Each photograph included a full-body person (female or male), facing either left or right. In addition, red discs were displayed on one or both of the left/right sides of each scene, ranging from zero to three discs. The number and position of discs changed in each trial, thus nine versions of each scene were constructed (depicting either: one, two, or three discs in front of the person; one, two, or three discs behind the person; one disc in front and one disc behind the person; two discs in front and one disc behind the person; or one disc in front and two discs behind the person), plus one “filler” version with no discs. On half of the experimental trials, self and other perspectives were consistent (i.e., the participants could see the same number of discs as the person in the scene), and on the other half of trials, self and other perspectives were inconsistent (i.e., the person in the scene could not see the some of the discs that were visible to participants). See Figure 1 for examples of these visual stimuli.

Since the red discs were superimposed on the visual scenes, we conducted a brief post-test to test the realism of our stimuli (i.e., that observers genuinely think the person in the scene could see the red discs when they are in front of them). Thirty participants on the Prolific.co online survey platform were presented with 51 scenes from our experiment, of which 34 showed a disc in front of the person and 17 showed a disc behind the person. Their task was to decide for each image whether the person can see the red disc or not. Results showed that for scenes depicting a disc in front of the person, participants agreed that the person could see the disc on average 74% (*SD* = 11%) of trials (vs only 0.2% for scenes that depicted a disc behind the person). Ratings varied between images (range: 4%–100%), which suggests that the visibility of the disc for the person in some scenes was limited. To account for this between image variability, image was included as a random effect in all statistical models.

### Procedure

The experiment was divided into two parts. In the first part, participants had to verify the number of discs that were visible according to their own perspective (self-perspective condition); the person in the scene’s perspective was never probed or mentioned. In the second part, participants had to verify the number of discs that were visible according to their own perspective (self-perspective condition) or according to the person in the scene’ perspective (other-perspective condition).

**Figure 2.**
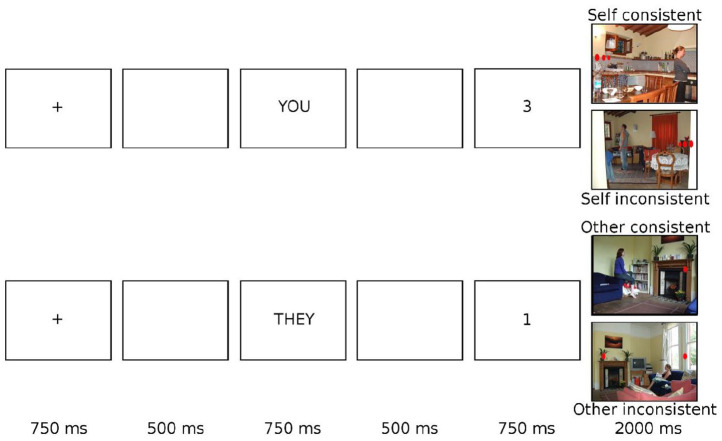
Schematic trial sequence of visual displays presented to participants in the visual perspective-taking task.

In both parts of the experiment, trials began with a fixation cross in the centre of the screen for 750 ms. Following a blank screen inter-stimulus interval (ISI) of 500 ms, a perspective cue was presented for 750 ms (always the word “YOU” in part 1, and either the word “YOU” or “THEY” in part 2). This indicated whether participants should answer according to their own or the person in the scenes’ perspective. Following a second blank screen ISI of 500 ms, a digit between zero and three was shown in the centre of the screen for 750 ms. Finally, a photograph of a natural scene (603 × 452 pixels) appeared in the centre of the screen, and participants were instructed to judge whether the number of discs in this image matched the preceding digit according to the cued perspective or not. Participants responded using keys “z” and “m” (key associations were counterbalanced across participants) as quickly and accurately as possible. The task moved to the next trial once a keyboard response had been detected or after a maximum response period of 2000 ms. See Figure 2 for examples of the trial sequence in each condition.

Trials could be either matching or mismatching. On matching trials, the number of discs that could be seen from the cued perspective in the photograph correctly corresponded to the preceding digit. On mismatching trials, the number of discs that could be seen from the cued perspective in the photograph did not correctly correspond to the preceding digit. Since mismatching trials require different processing (Samson et al., 2010), only matching trials were analysed.

The experiment started with a practice block of 13 trials, in which participants only responded according to their own perspective. Next, they completed two blocks of self-perspective only trials, with 52 trials in each block. The second part of the experiment was preceded by a second practice block of 26 trials and was composed of four blocks that mixed self- and other-perspective trials, with 52 trials in each block. Each experimental block included 24 matching trials, 24 mismatching trials, and four “filler trials” (where no discs were present on the photographs so that the disc number zero was sometimes correct even for self-perspective trials). Of these, half were consistent trials, where the avatar and participant saw the same number of discs on the wall, and half were inconsistent trials, where the avatar and participants’ views were different. Trials were presented in a random order. Each scene was repeated seven times during the experiment, always with a different combination of red discs, perspective cue and number prompt. The full experiment lasted approximately 60 min.

In sum, in the first part of the experiment (only self-perspective condition) one variable was manipulated (Consistency: consistent vs inconsistent) in a within-subjects design. In the second part of the experiment (mixed self- and other-perspective conditions), two variables were manipulated in a 2 (Consistency: consistent vs inconsistent) × 2 (Perspectives: other vs self) within-subjects design. The dependent variables were reaction time and accuracy.

## Results

All analysis procedures were pre-registered, and the full datasets and analysis scripts are available on the Open Science Framework web pages (https://osf.io/tn6dw/). Statistical analyses were conducted in R version 4.0.1. Following Samson et al.’s (2010) procedure, we investigated the factors affecting participants’ accuracy and response times only in matching trials, and reaction times were calculated based on correct responses only. Separate analyses were conducted for part 1 (self-perspective condition) and part 2 (mixed self- and other-perspective conditions) of the experiment.

In part 1, linear mixed models were used to test the effect of consistency (consistent vs inconsistent) on accuracy and response times. Each model included the fixed effect of consistency (contrast coded, −.5 vs .5), random effects for participants and image, and a random slope for consistency (as suggested by the maximal random effects structure, Barr et al., 2013). If the model failed to converge, we simplified the model by removing the random effect until the convergence was reached. In part 2, linear mixed models were used to test the effects of perspective (self vs other) and consistency (consistent vs inconsistent) on accuracy and response times. Each model included fixed effects for perspective and consistency (both contrasts coded, −.5 vs .5), random effects of participants and image, and crossed random slopes for perspective and consistency (as suggested by Barr et al., 2013). If the model failed to converge, we simplified it by removing the more complex random effect until the convergence was reached.

### Part 1: self-perspective condition

**Figure 3.**
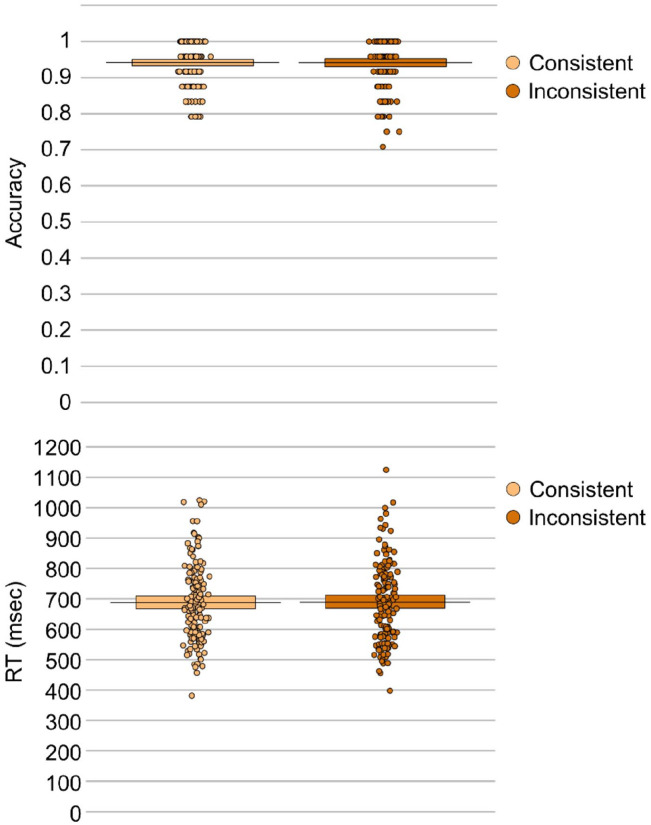
Mean response accuracy (top panel) and response times (bottom panel) for each consistency condition in the self-perspective condition. The plots show raw data points, a horizontal line reflecting the condition mean, and a rectangle representing the Bayesian highest density interval.

The effect of consistency was non-significant on both accuracy, β < –0.01, *SE* = 0 .01, *t* = 0.02, *p* = .984, and response times, β = –4.71, *SE* = 23.16, *t* = 0.20, *p* = .840. Thus, consistent trials (*M_ACC_* = 0.94, *M_RT_* = 688.12 ms) did not differ from inconsistent trials (*M_ACC_* = 0.94, *M_RT_* = 688.23 ms) when participants were only prompted to respond according to their own perspective (see Figure 3).

### Part 2: mixed self- and other-perspective conditions

Analysis of accuracy revealed effects of consistency, β = –0.10, *SE* = 0.01, *t* = 16.73, *p* < .001, and perspective, β = 0.03, *SE* = 0.01, *t* = 4.75, *p* < .001. Accuracy was higher for consistent compared to inconsistent trials (*M* = 0.97 vs 0.87, respectively), and for self-perspective compared to other-perspective trials (*M* = 0.94 vs 0.91, respectively). An interaction between perspective and consistency was also present, β = 0.05, *SE* = 0.01, *t* = 4.11, *p* < .001, reflecting significantly higher accuracy on consistent versus inconsistent trials for both self (β = –0.08, *SE* = 0.01, *t* = 8.28, *p* < .001) and other (β = –0.13, *SE* = 0.01, *t* = 16.11, *p* < .001) perspectives, though the consistency effect was larger when participants were cued to take the other-perspective compared to when they were cued to take the self-perspective, *M_DIFF_* = 0.13 vs 0.08, respectively; *t*(149) = 3.72, *p* < .001, *d* = .31.

Analysis of response times also revealed an effect of consistency, β *=* 96.54, *SE* = 9.88, *t* = 9.77, *p* < .001, due to faster responses on consistent compared to inconsistent trials (*M* = 670.28 ms vs *M* = 763.71 ms). Neither the effect of perspective, β = 4.01, *SE* = 9.88, *t* = 0.41, *p* = .686, or the interaction between perspective and consistency was significant, β = –36.96, *SE* = 19.77, *t* = 1.87, *p* = .065. Follow-up analyses showed that both self (β = 78.23, *SE* = 15.18, *t* = 5.15, *p* < .001) and other (β = 115.32, *SE* = 12.71, *t* = 9.08, *p* < .001) perspectives elicited significantly faster responses on consistent versus inconsistent trials, and this consistency effect was larger when participants were cued to take the other-perspective compared to when they were cued to take the self-perspective, *M_DIFF_* = 105.56 ms versus 81.31 ms; *t*(149) = 2.67, *p* = .008, *d* = .22.

**Figure 4.**
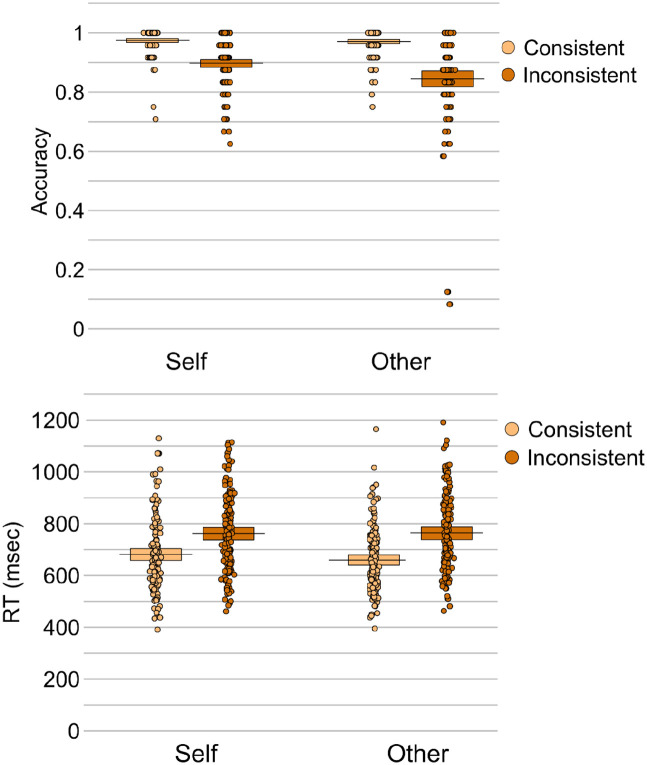
Mean response accuracy (top panel) and response times (bottom panel) for each condition in the mixed self- and other-perspective conditions. The plots show raw data points, a horizontal line reflecting the condition mean, and a rectangle representing the Bayesian highest density interval.

Taken together, the accuracy and response time data converge to show that participants experienced both altercentric and egocentric interference, though the egocentric interference was greater than the altercentric interference (see Figure 4).

## Discussion

In this article, we sought to investigate the cognitive mechanisms that underlie Level-1 VPT, specifically testing the extent to which egocentric and altercentric biases persist when VPT is tested in complex real-world environments. Participants completed a version of Samson et al.’s (2010) dot-probe VPT task, adapted to use photographs of real people in complex natural scenes, rather than the computer simulations of avatars in blank rooms that have been used previously. They had to verify the number of discs in each visual scene according to their own or the other person’s visual perspective; on some trials the two perspectives were inconsistent (i.e., each saw a different number of discs), while in others they were consistent. To examine the automaticity of any altercentric interference, participants first completed the task only for self-perspective trials (since this tested implicit other perspective-taking, without carry-over effects from being explicitly prompted to consider the other perspective on some trials). Participants’ behavioural responses on the task (i.e., response accuracy and reaction times) were recorded.

Results revealed no difference in accuracy or reaction times when consistent and inconsistent trials were tested for the self-perspective in isolation, suggesting that participants did not automatically compute the altercentric perspective when the other person’s perspective was task-irrelevant. These results therefore go against the predictions of a cognitively efficient implicit mentalizing view, which suggests that humans are automatically sensitive to social information in their environment, and that the brain cannot ignore other peoples’ perspectives even when they are irrelevant (Samson et al., 2010). Nevertheless, it is notable that previous studies that have tested the self-perspective in isolation using more simple stimuli have found significant effects of consistency (e.g., Ferguson et al., 2018, Experiment 3; Samson et al., 2010, Experiment 3; Santiesteban et al., 2014, Experiment 2). These studies differed from the current design by using more simple stimuli, with a centrally-located avatar on a plain background, which may have increased the salience of the avatar’s perspective. This suggests that the complex natural scenes used in the current experiment increased the cognitive demands of the task and prevented spontaneous perspective-taking, perhaps by disrupting domain-general processes, including those that mediate automatic attentional orienting, and that this prevented or delayed attention capture of the person’s face and subsequent perspective-taking.

In contrast, when self- and other-perspective conditions were mixed, both egocentric and altercentric biases were clear; accuracy was reduced, and reaction times were slower when the two perspectives were inconsistent compared to when they were consistent. Thus, when the person in the scene was a real person rather than a computer-generated avatar, participants still could not resist inferring their own perspective and experienced interference from this self-perspective even on trials where they were prompted to respond according to the other person’s perspective. Similarly, when participants were explicitly prompted to consider the other perspective on some trials, this increased the salience of this other person’s perspective such that they inferred the other perspective, even on trials where they were cued to take their own perspective and it was not necessary for them to calculate the other perspective. This shows that the lack of consistency effect in self-only trials cannot be explained merely by a disruption to bottom-up perceptual discrimination of the person due to our complex visual scenes.

In addition, when the perspective trials were mixed, the consistency effect was larger for other- than self-perspective conditions, reflecting greater egocentric interference compared to altercentric interference. These results are consistent with the many previous studies that have employed this task and reported consistency effects for both self- and other-perspective conditions when these trial types were mixed and the other perspective was explicitly salient through prompts to take the avatar’s perspective on some trials (e.g., Capozzi et al., 2014; Ferguson et al., 2017; Furlanetto et al., 2016; Nielsen et al., 2015; Qureshi et al., 2010), and also with studies that have shown a preference to attend to faces/people in visual scenes (e.g., Bindemann & Burton, 2008; Bindemann et al., 2007, 2010; Brown et al., 1997; Dupierrix et al., 2014; Farroni et al., 2005; Hershler & Hochstein, 2005; Mayer et al., 2015; Olk & Garay-Vado, 2011). Increasing participants’ awareness of another person’s knowledge (not simply their presence) has also been shown to enhance level-2 perspective-taking, which is traditionally assumed to require more effortful processing. Recent research has revealed that aspectual properties of another person’s perspective can be calculated spontaneously when participants engage in a collaborative task with the other person (Surtees et al., 2016), or when the avatar’s awareness of the objects around them (i.e., not only their visibility) is cued (Elekes et al., 2016). In our study, consistency effects in the mixed self- and other-perspective trials reinforce the proposal that other peoples’ perspectives can be accommodated spontaneously (i.e., rapidly and when relevant) when top-down contextual cues highlight the avatar’s perspective, even in complex real-life environments where bottom-up stimulus features increase the cognitive demands of the task. This pattern could be explained in terms of both mentalizing and directional orienting influences.

Despite the current study’s enhanced ecological validity, further questions remain about the role of visual context in VPT. For example, our study only included one person in each scene, while in everyday life we encounter multiple people simultaneously; it remains unknown whether others’ visual perspectives are adopted at all in these multi-party scenarios, and if so, how the different perspectives are prioritised for processing. Similarly, though both the mentalistic and the selective attention interpretations acknowledge that other perspective-taking is subject to limits with increasing complexity, there is currently no specificity on what this limit should be, and it is likely that these cognitive demands would be pushed even further in more dynamic scenes or interactive tasks. In addition, our stimuli were 2D photographs of unfamiliar people in unfamiliar environments. Future research should manipulate social contexts further in fully immersed environments (e.g., using virtual reality or real-life) to explore the impact of these factors individually.

In conclusion, the current study suggests that VPT can be activated spontaneously in real-world contexts, but is influenced by various contextual cues from bottom-up perceptual features of the stimulus and top-down cues that enhance the salience of the avatar’s perspective. Given the range of complex environments and interaction situations that we engage in during everyday life, it seems unlikely that processes for mentalizing or directing attention would be activated automatically, regardless of need, since this would be too cognitively demanding. Instead, our results support the distinction between automatic and spontaneous perspective-taking proposed by O’Grady et al. (2020), in which other peoples’ perspectives can be inferred rapidly and unconsciously, provided that this computation is in line with specific goals (e.g., a motivation to understand others, Carruthers, 2015) or contextual cues (e.g., Todd et al., 2017). We have identified a number of salient cues that highlight the importance or relevance of the other person’s perspective in this VPT task, and that prompt spontaneous orienting to other peoples’ perspectives. In line with previous studies, these include tasks that use explicit prompts to take the other person’s perspective on some trials (as in our mixed self- and other-perspective blocks), or even trial-by-trial cues to take one’s own perspective during a self-only VPT task (since this highlights the contrast between the self and other). Importantly, we have shown that increasing the complexity of the visual environment can disrupt level-1 VPT in the absence of top-down cues by reducing the salience of the avatar/person and interfering with attentional orienting processes which input to mentalizing processes.

## Notes

1. We note that Langton (2018) used photographs of real people in a visual perspective-taking task; however, the scenes did not depict complex naturalistic environments from real-life. Instead, participants had to judge the visual perspective of a person sitting in the corner of a plain white-walled room. Responses were faster when participants could see the same number of dots as the person, suggesting that altercentric interference is not diminished when the computer-generated avatar is replaced by a real person.
